# Predictors of fracture healing in patients with recalcitrant nonunions treated with autologous culture expanded bone marrow‐derived mesenchymal stromal cells

**DOI:** 10.1002/jor.24184

**Published:** 2019-01-29

**Authors:** Atanu Bhattacharjee, Jan H. Kuiper, Sally Roberts, Paul E. Harrison, Victor N. Cassar‐Pullicino, Bernhard Tins, Stefan Bajada, James B. Richardson

**Affiliations:** ^1^ The Robert Jones and Agnes Hunt Orthopaedic Hospital NHS Foundation Trust Oswestry UK; ^2^ Institute for Science and Technology in Medicine Keele University Keele UK

**Keywords:** bone, biomaterials, progenitors and stem cells, bone biology, bone tissue engineering and repair, bone fracture

## Abstract

The study reports the prospective outcome of treating severe recalcitrant fracture nonunion in patients with autologous bone marrow‐derived mesenchymal stromal cells (BMSC) from 2003 to 2010 and analyze predictors of union. Autologous BMSC were culture expanded and inserted at nonunion site with or without carriers in addition to surgical stabilization of the fracture. Radiological union was ascertained by musculoskeletal radiologists on plain radiographs and/or CT scans. A logistic regression analysis was performed with cell‐expansion parameters (cell numbers, cell doubling time) and known clinical factors (e.g., smoking and diabetes) as independent variables and fracture union as the dependent variable to identify the factors that influence bony healing. An Eq5D index score assessed the effect of treatment on general quality of health. A total of 35 patients (mean age 51+/−13 years) with established nonunion (median 2.9 years, 1–33) and, at least one failed nonunion surgery (median 4,1–14) received treatment. Fracture union was achieved in 21 patients (60%; 95%CI 44–75) at 2.6 years. Multiple penalized logistic regression revealed faster cell doubling time (*p* = 0.07), absence of diabetes (*p* = 0.003), less previous surgeries (*p* = 0.008), and lower age at cell implantation (*p* = 0.02) were significant predictors for fracture union. A significant increase in Eq5D index (*p* = 0.01) was noted with a mean rise of the score by 0.34 units (95%CI 0.11–0.58) at 1 year following the study. In summary, the study revealed cell doubling time as a novel in vitro parameter in conjunction with age, multiple surgeries, and diabetes as being significant predictors of the fracture union. © 2018 The Authors. *Journal of Orthopaedic Research*® Published by Wiley Periodicals, Inc. J Orthop Res 37:1303–1309, 2019.

A total of 850,000 new fractures are reported annually in the UK. The majority progress to uncomplicated healing, except in 5–10% of patients whose fracture does not heal.^1^ Recent Scottish data estimates the overall annual incidence of nonunions at 19 per 100,000 people, equivalent to the incidence of revision hip replacements.^1^, ^2^ However, nonunion in young individuals can have a devastating impact on the patient's life for longer. Lower limb nonunions make up 6.7 per 100,000 people, with a treatment cost per person of $11,500 to $132,000 to the National Health Service in the UK.^2^

The haematoma which forms post‐fracture in humans contains cells which have multi‐lineage differentiation potential including the capacity for osteogenic differentiation; the phenotype of these cells is similar to mesenchymal stem cells (MSC).^3^, ^4^ However, nonunion stromal cells (NUSCs) grown from human nonunion sites were noted to have slower doubling time, lower osteogenic potential, and a higher rate of apoptosis in comparison to bone marrow derived MSCs from healthy donors.^5^ These NUSCs were also found to secrete Dickkopf‐1(Dkk‐1) which is an antagonist to the Wnt signalling pathway and inhibits osteogenic differentiation and fracture healing.^5^

Hence, exploring autologous osteoprogenitor cells remote from the site of the nonunion will be an intutive choice. Successful regeneration of large bone defects by using in vitro expanded autologous BMSC and subsequently re‐implanting them in scaffolds has been reported in one case series of four patients.^6^ Given that in vitro studies have demonstrated no difference in the osteogenic capacity of BMSC from iliac crest in patients with atrophic nonunions and healthy volunteers,^7^ one could use a similar approach in cases of nonunions. Indeed, case series have reported success using autologous bone marrow derived cells from iliac crest for the treatment of nonunions, implanted either after in‐vitro culture expansion^8^, ^9^ or as a concentrate produced immediately after harvest.^8^, ^9^ Given these promising results, we decided to investigate the utility of using culture‐expanded autologous BMSCs to treat patients with a “recalcitrant” nonunion, which is characterized by persistence of established nonunion (as defined by the US Food and Drug Administration (FDA) for at least 1 year, in addition to at least one previous failed surgery to treat the nonunion. An additional aim was to determine baseline predictors of successful bone healing, which could help to target the therapy or assess the potency of the cells.

## METHODS

Patients with recalcitrant nonunion were invited to participate in the study of evaluating the efficacy of autologous BMSC in achieving fracture union.

Patient inclusion criteria were:

- Nonunion following fracture of the tibia or femur.
- An established nonunion according to the US FDA criteria^10^ for at least 1 year.
- At least one failed previous surgery for nonunion.

Patient exclusion criteria were:

- Skeletal immaturity.
- Pregnant or breastfeeding.
- Nonunion following pathological fractures.
- Infection during BMSC culture.

### In Vitro BMSC Culture

Bone marrow aspirates from the iliac crest of the patients with nonunion were harvested with aseptic precaution in the theatre. A Jamshidi needle attached to a heparinized syringe (Becton Dickson Medical Supplies, Cowley, UK) was used to aspirate between 2 and 10 ml of bone marrow (with a new insertion site after each 2 ml). The BMSC's were isolated and cultured from the aspirate according to the previously published protocol from our centre.^11^

These cells were plated at a density of 2 × 10^7^ cells per 250 ml flask (Polystyrene Tissue Culture Flask, BD Biosciences, Cowley, Oxford, UK) with 20 ml of the DMEM‐F12 10% FCS and antibiotics (Penicillin & Streptomycin). After 24 h the non‐adherent cells and medium were removed and the flask washed with PBS. The adherent cells were continued to be cultured in monolayer with the same medium at 37°C, 5% (v/v) CO_2_ until it reached 70% confluence. Then the cells were passaged by trypsinization and re‐seeded at a density of 5 × 10^3^ cells/cm^2^. Cell numbers were counted by trypan blue exclusion at the end of each passage and the doubling time calculated over a period of 3 weeks. The discarded medium was sent to the microbiology department for infection screening. At 3 weeks, cells were harvested (usually passage 3) culture and suspended in pure autologous serum before being transported to the operating theatre for insertion at the nonunion site by the surgeon.

### Surgical Technique

The nonunion site was exposed and decorticated to allow implantation of the cells. Cells in autologous serum was mixed with carriers in 27 patients (β tri‐calcium phosphate (Allogran®‐R, Biocomposites, Keele, UK), calcium sulphate (CaSO_4_; Stimulan®, Biocomposites, Keele, UK), hydroxyapatite (Allogran® N, Biocomposites), or a combination of β tri‐calcium phosphate and calcium sulphate) and left for 10–15 min. This allowed attachment of cells to the carrier and subsequently the mixture was inserted at the nonunion site. The same process occurred for the remaining eight patients, but without the use of carriers; rather cells suspended only in autologous serum were applied directly to the nonunion site. Surgical stabilization of fracture was undertaken, if required, however not routinely carried out.

### Outcome Measures

Radiological evidence of fracture union at follow‐up as assessed by independent specialized musculoskeletal radiologists was used as a primary outcome criterion for the bony healing of the nonunion site. Any further interventions for fracture fixation due to the persistence of nonunion was defined as failure of the treatment, and the fracture was deemed not united by the study intervention. Surgical interventions for wound complications, pin‐tract infection following application of external fixation device, revision of external fixator pins due to local soft‐tissue complications, dynamization, or revision of interlocking screws following intramedullary fixation were not considered as treatment failures. The change in the EQ‐5D index at 1 year was also used as an outcome measure to assess the impact of treatment on the general quality of life in these patients. Safety was assessed from the occurrence of any postoperative serious adverse events until final follow‐up. The influence of six continuous (age at fracture, time since original fracture, number of previous operations, age at cell implantation, number of implanted cells, cell doubling time) and 11 categorical (gender, fracture site, type of fracture, type of nonunion, presence of infection at cell insertion, diabetes, alcohol usage, smoking, previous bone graft treatment, previous BMP treatment, carrier type) potential baseline and treatment‐related predictors of fracture union was analyzed.

### Statistical Methods

Fisher's exact test was used to identify univariable categorical predictors of fracture union at the nonunion site. Penalized logistic regressions were used to identify continuous variables. Multiple penalized logistic regressions were used in a multivariable analysis to determine if combined independent variables gave a better prediction. For this analysis, all univariable predictors with *p* < 0.25 were considered potential candidates for inclusion in the multivariable model.^12^ Nagelkerke's *R* ^2^ was used as an overall measure of model performance.^13^

A multilevel model with a random intercept was used to determine the difference in the EQ‐5D index before and 1 year after treatment. This method was chosen to include all patients, even when one of their pre or postoperative EQ‐5D score was missing.

Statistical analyses were performed using *R* version 3.0.2, using the packages “logistf” and “nlme.” A two‐sided *p*‐value below 0.05 was assumed to denote statistical significance.

## RESULTS

### Patient Demographics

A total of 37 patients were invited to participate in the study; one patient did not meet the inclusion criteria, and one patient declined participation in the study. Hence, 35 patients (21 males, 14 females), with a mean age of 50.6 years (range 17–75) at the time of treatment, were recruited (Table 1). The median duration of established nonunion was 2.9 years (range 1–33); patients had undergone a median of four surgical interventions (range 1–24) before cell insertion at the fracture site. Each patient in this cohort met the criteria defining “recalcitrant” nonunions of fracture. Twenty‐nine patients had atrophic nonunions, whereas six had hypertrophic nonunions; 19 patients had femoral, and 16 had tibial fracture nonunions. There were no dropouts or loss to follow‐up during the first 12 months except one patient who died from unrelated causes 3 months after the study intervention.

**Table 1:** Baseline Demographics and Clinical Characteristics

Parameter	Value
Demographics	
Sex	Male 21 (60%), female 14 (40%)
Age at accident (years)	Mean 45.2 (SD 12.4; range 16–71)
Age at cell implantation (years)	Mean 50.6 (SD 12.5; range 17–75)
Time from accident to cell implantation (months)	Mean 56.4, median 35 (range 12–396)
Fracture and nonunion characteristics	
Site	Femur 19 (54%), tibia 16 (46%)
Velocity	High 20 (57%), low 15 (43%)
Open or closed	Open 18 (52%), closed 13 (37%), Unknown 4 (11%)
Atrophic or hypertrophic	Atrophic 29 (83%), hypertrophic 6 (17%)
Number of operations before cell implantation	Mean 2.8, median 2 (range 1–14)
Number of cases with previous autologous bone graft or BMP	Graft 10 (29%), BMP 0, Both 2 (6%)
Comorbidities	
Smoking	Yes 8 (23%), No 27 (77%)
Alcohol	Yes 16 (46%), No 13 (37%), Unknown 6 (17%)
Diabetes mellitus	Yes 5 (14%), No 30 (86%)

### In Vitro BMSC Culture

Autologous BMSC's were culture expanded for three weeks with a mean cell doubling time of 7.2 days (range 2–31, SD‐6.24) and without any evidence of infection in the media. A mean of 5.5 × 10^6^ BMSCs (range 2–10 × 10^6^, SD 1.99 × 10^6^) was inserted into the nonunion site. Twenty‐seven patients (77%) received a carrier based on β‐TCP, calcium sulphate or a combination of both or hydroxyapatite (Table 2). For eight patients (23%) autologous serum alone was used without a carrier.

**Table 2:** Overview of Carriers Used in the Study

Carrier type	Number of cases (%)
β tri calcium phosphate with calcium sulphate	16 (46%)
β tri calcium phosphate	6 (17%)
Calcium sulphate	4 (11%)
Hydroxyapatite	1 (3%)
Serum	8 (23%)

### Fracture Union Rate and Predictors of Union

**Figure 1:**
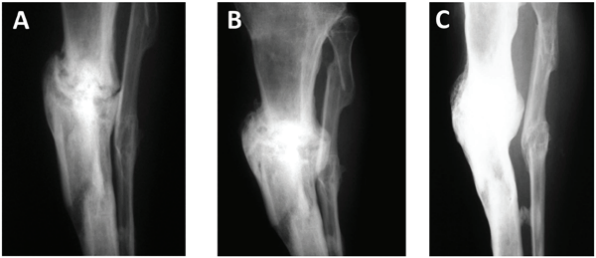
Example of a patient whose fracture healed. The patient was a 42 year old male who fractured his tibia in a road traffic accident 8 years before cell implantation. In those 8 years, the fracture had four previous interventions. A: Pre‐cell implantation. B: Four months after implanting 5 × 106 BMSCs in a CaS carrier. C: 12 months after cell implantation the fracture has united.

A total of 21 out of 35 patients (60%; 95%CI 44–75%) achieved radiological fracture union at an average follow‐up of 2.6 years (range‐0.24–8.24) ascertained by specialist musculoskeletal radiologists in a orthopaedic tertiary care unit (Fig. 1). The vast majority of categorical independent variables, including the type of carrier, had a small but non‐significant effect on the union rate (Table 3). The effect of diabetes did not reach significance, (*p* = 0.06), but reduced the odds of achieving union by over eight‐fold (Table 3). Of the five patients with diabetes, four failed to reach fracture union. Among the continuous predictors, the number of previous operations and the cell doubling time during in‐vitro culture of BMSCs were significant predictors of the fracture union (Table 4).

**Table 3:** Univariable Analysis of Categorical Predictors of Union

Factor	OR (95% CI)	*p*‐value
Gender (Male)	1.4 (0.27–7.0)	0.73
Fracture site (tibia)	1.7 (0.35–9.2)	0.50
Type of fracture (open)	0.82 (0.15–4.4)	1.0
Type of nonunion (hypertrophic)	3.6 (0.34–192)	0.37
Infection at insertion	7.46 (0.43 to 129)	0.48
Alcohol	1.1 (0.16–7.1)	1.0
Diabetes	0.12 (0.0022–1.4)	0.06
Smoking	0.39 (0.046–2.8)	0.39
Previous bone graft treatment	0.40 (0.06 to 2.3)	0.27
Previous BMP treatment	0.60 (0.06 to 2.3)	1.0
Carrier type	‐	0.61

OR is Odds Ratio, CI is Confidence Interval. *p*‐values determined using Fisher's exact test.

**Table 4:** Univariable Analysis of Continuous Predictors of Healing

Factor	Union (median, IQR)	Non‐union (median, IQR)	OR (95%CI)*	Nagelkerke's *R* ^2^*	*p*‐value*
Age at accident	42.4 [37.7, 50.6]	53.9 [39.8, 57.3]	0.97 (0.91 to 1.02)	0.06	0.25
Years since accident to cell implantation	2.3 [1.5, 3.9]	3.3 [2.8, 5.1]	0.92 (0.63 to 1.03)	0.08	0.17
Number of previous operations	2.0 [2.0, 3.0]	3.0 [2.0, 5.0]	0.58 (0.29 to 0.95)	0.22	0.02
Age at cell implantation	47.8 (14.1)	55.0 (8.8)	0.96 (0.89 to 1.01)	0.11	0.12
Cell number (10^6^)	5.5 [5.0, 6.5]	5.0 [4.0, 6.0]	1.1 (0.78 to 1.6)	0.01	0.56
Cell doubling time (days)	5.1 [3.9, 6.0]	7.0 [3.7, 12.7]	0.87 (0.68 to 0.99)	0.20	0.04

IQR is Interquartile Range, OR is Odds Ratio, CI is Confidence Interval. OR and their 95%CI, Nagelkerke's *R* ^2^ and *p*‐values determined using penalized logistic regression

In the multiple penalized logistic regression analysis, we used all four univariable predictors with *p* < 0.25 as potential predictors, namely having diabetes, the number of previous surgical interventions, age at cell implantation, and cell doubling time. Age at accident and years since accident were excluded due to their strong correlation with age at implantation (Spearman's rho = 0.86; *p* < 0.001) and number of previous operations (Spearman's rho = 0.60; *p* < 0.001), respectively. According to the regression model, the chance of union was larger in patients with a lower age at cell implantation, fewer previous surgical interventions, cells with a shorter doubling time and non‐diabetic patients (Table 5). The model explained approximately 90% of the variation in outcome (Nagelkerke's *R* ^2^ = 0.90).

**Table 5:** Results of the Multiple Logistic Regression Analysis

Factors	Coeff. (95%CI)	OR (95%CI)	*p*‐value
Age at cell implantation	−0.15 (−0.36 to −0.02)	0.86 (0.69 to 0.98)	0.02
Number previous interventions	−0.48 (−2.3 to −0.11)	0.62 (0.097 to 0.90)	0.008
Doubling time	−0.19 (−0.63 to 0.01	0.82 (0.53 to 1.01)	0.07
Diabetes	−4.9 (−11 to −1.5)	0.008 (3 · 10^−5^ to 0.24)	0.003

CI is Confidence Interval, OR is Odds Ratio. Coefficients, ORs and *p*‐values determined using penalized logistic regression. For this model, Nagelkerke's *R* ^2^ was 0.90.

### Adverse Events

One patient developed sepsis following implantation of BMSC requiring intensive hospital care. The patient recovered uneventfully without overt residual infection. Two patients died before final follow‐up, but apparently due to causes unrelated to the nonunion treatment. One died within 3 months of the intervention due to an aorto‐enteric fistula, and the other died 3 years after the intervention due to heart failure on a background of tricuspid regurgitation. Another patient was reported to have a benign gastric tumor 6 years after the BMSC insertion. The tumor was excised, and the patient was doing well during the latest follow‐up at 9 years.

### Health‐Related Quality of Life

The mean preoperative EQ‐5D utility index was very low (0.09; Table 6). The score increased significantly by 0.34 at 1 year (*p* = 0.01), with no evidence of a difference in benefit between patients who had and who had not achieved union (*p* = 0.14 for interaction between union and slope). An analysis of the five dimensions of the EQ‐5D showed noticeable improvement in scores pertaining to anxiety/depression, pain/discomfort, and usual activity, but minimal differences in mobility and no changes in self‐care at 12 months.

**Table 6:** Change in EQ‐5D at 1 Year From Cell Implantation

Outcome	Mean pre‐op (SD)	Mean post‐op (SD)	Difference^*^ (95%CI)	*p*‐value
EQ‐5D index	0.09 (0.45)	0.32 (0.41)	0.34 (0.11 to 0.58)	0.01

^*^ Difference calculated using random intercept multilevel model. This difference does not equal the difference between the pre‐op and post‐op columns but does provide a better estimate by properly accounting for repeated measures.

## DISCUSSION

In our study, 60% of patients with a recalcitrant nonunion of fracture and very varied often complex histories, achieved radiological union. Faster cell doubling time, the absence of diabetes, fewer previous surgeries, and lower age at cell implantation were significant predictors for bony healing in these patients. The overall union rate of 60% may seem low compared to union rates in other studies of biological enhancement treatments for lower limb fracture non‐unions, which typically range from 88% to 94% (Table 7).

**Table 7:** Comparison of Baseline Patient Characteristics and Union Rates Between Four Reports of Biological Nonunion Treatments

Study	Treatment	Age (Mean, range)	Previous surgeries (mean, range)	Months since injury (mean, range)	Diabetes (number/total, %)	Clinical union rate (%)
Current	Culture‐expanded BMSCs	50.6 (17–75)	2.8 (1–14)	56 (12–396	5/35 (14%)	21/35 (60%)
Pneumaticos et al.^22^	Autologous bone graft and/or BMP‐7	38.8 (17–78)	2.0 (0–11)	24.0 (6–317)	Nd	94.3%
Kanakaris et al.^17^	BMP‐7	42.6 (19–78)	2.5 (0–11)	23 (9–317)	1/60 (2%)	61/68 (89.7%)
Hernigou et al.^12^	Percutaneous bone marrow injection	40.0 (18–78)	1.1 (0–2)	8.7 (6–12)	3/60 (5%)	53/60 (88%)

The mean values cited for Pneumaticos et al., a meta‐analysis, are the pooled means of all 13 studies in that review reporting the characteristic; nd is not documented

However, our case series represents a group of patients defined to have severe “recalcitrant” nonunions, for many of whom the next considered treatment would be amputation. On average, the patients in our study were around 10 year older, had one extra previous surgery and 32 months more had passed since their original fracture than typically in other series (Table 7). Based on our analysis (Table 5), the higher age and extra surgery would reduce the odds of healing five‐fold. Moreover, our study included a three to five times larger proportion of patients with diabetes than other studies (Table 7). Diabetes was associated with a very poor prospect of healing in our study, and accumulated evidence from animal studies suggests that bone regeneration is strongly reduced in animals with diabetes.^14^ Taken together, these differences probably explain why the overall healing rate in our study was lower than others’. It also indicates that culture‐expanded BMSCs are no “magic bullet” for certain patients with recalcitrant non‐unions, and better solutions are still needed for those more elderly patients with diabetes, for example.

The study also shows that longer in vitro cell doubling time during culture expansion of BMSC was related to reduced likelihood of achieving fracture union, whereas the number of cells implanted had no effect. Doubling time was reported critical in a study comparing osteogenic potentials of culture expanded human BMSCs.^15^ The study found considerable donor variations of in vivo osteogenic potential and identified a longer cell doubling time during in vitro expansion as the best predictor of poor osteogenicity.^16^ Hence, our data might also help define future quality criteria for BMSCs in bone regeneration, with the cell doubling time of patients’ BMSCs as a potential biomarker to predict their odds of achieving union before treatment. The lack of relevance of cell numbers seems to be in contrast to the study of percutaneous bone marrow grafting in which larger numbers of injected cells increased the odds of fracture union.^9^ However, the smallest number of implanted cells in our study is two million (range 2–10 million) which significantly exceeds even the largest number of cells used in that study (70,000). There remains no clear optimal number of cells to implant for other cell therapies, for example, in cartilage repair procedures used in the clinic.^16^

Our study also found that three clinical or demographic factors (no diabetes, lower age, and fewer previous operations) predicted the chance of union. As mentioned above, diabetes mellitus and also age are well‐known risk factors contributing to nonunions^17^ and perhaps their persistence. The number of previous treatments characterizes the persistence of a nonunion, and therefore, its emergence as a risk factor for failure to heal is expected. The injury mechanism or type of fracture (“open” vs. “closed”) were not significant predictors of nonunion, in agreement with the findings of the SPRINT trial for tibial fractures.^18^

The unrelated deaths and tumor in the present study is in line with others; for example, a report on the safety of stem cells for orthopaedic regeneration reported a tumor (of the liver) in their series, which was likewise considered unrelated.^19^ The lack of related serious adverse events in our study highlights the safety of using autologous in vitro expanded BMSCs to treat nonunions. BMSCs in this study were grown for a maximum of three passages to minimize the risk of genetic changes associated with long‐term culture and expansion.^20^ Cells were cultured in autologous serum, removing risks of prion or virion transmission from bovine‐derived serum.^20^ In addition, the cells were implanted locally rather than administered intravenously, reducing risks of aberrant remote proliferation.^21^

Twelve months after cell implantation in our study, the EQ‐5D index had improved significantly by 0.34 points, suggesting substantial overall improvement for this patient group. Despite this improvement, the EQ‐5D index remained relatively low, in line with a previous study of patients with fracture nonunions.^22^ This finding highlights the substantial influence of quality of life from a nonunion fracture.

The study is limited by being a report of a case series and lacks the robustness of randomization and evaluation of the outcome in a control group; in addition there was great variability in the patient group in terms of previous procedures, time since fracture etc. Were a trial to be conducted among a more homogenous group of patients presenting with fewer previous procedures, the effect of cells may be even greater. The use of four different types of osteoconductive carriers can also potentially influence the outcome of fracture union and such a variety of carriers may be considered a limitation. However, a subgroup analysis with the five groups of patients (according to carrier types, including one receiving no carrier) showed this factor contributed no significant difference to the clinical outcome.

The fact that there remained 40% of patients who did not achieve union of their fracture following cell therapy may indicate that this group of patients had conditions that were particularly unsuitable for this approach of treating with autologous cells. This could in part relate to the environment that the cells were being implanted into. Bajada et al.^3^ demonstrated that cells in the vicinity of the nonunion site secreted increased levels of the Wnt signaling inhibitor, DKK1. Since Wnt proteins promote BMP‐mediated osteoblastic differentiation, production of DKK1 in vivo could explain a decreased propensity for mineralization and bone formation at a fracture site. An alternative approach for promoting osteogenesis may be to select a sub‐group of mononuclear cells from the bone marrow; certainly, a Phase I/IIA trial, which used granulocyte colony stimulating factor (G‐CSF)‐mobilized peripheral blood CD34+ cells, has shown encouraging results in nonunion patients with healing in 71% of patients.^23^ A suggestion for their success is that these cells have the ability to differentiate into cells of not only osteogenic but also vasculogenic and haematopoietic lineages. In addition, if a particular patient has one of many genotypes which may lead to a lesser likelihood of osteogenesis, then perhaps any autologous therapy for nonunion is doomed to fail and the use of an allogeneic therapy would be beneficial in such individuals.

In conclusion, one cell characteristic (faster cell doubling during in vitro culture) and three patient characteristics (lower age, not having diabetes, and fewer previous operations) were strongly correlated with bone healing in patients with recalcitrant nonunion. Implantation of culture expanded‐BMSC to treat such nonunions was correlated with an improvement in the patients’ general quality of life and wellbeing. Further improvement in patient selection or perhaps the use of an alternative cell population, may aid outcomes in patients who remain difficult to achieve unions in, such as the more elderly patient and, or those with diabetes mellitus.

## AUTHORS’ CONTRIBUTION

A.B. is responsible for collection of the data, analysis, writing, and editing the paper. J.H.K. responsible for design, data analysis, proof reading, and editing. S.R. responsible for proof reading and editing. P.E.H. responsible for in vitro work, proof reading. V.N.C.‐P. responsible for radiological analysis. B.T. responsible for radiological analysis. S.B. responsible for in vitro work. J.B.R. (deceased) responsible for Design, senior operating surgeon delivering the study, proof reading, and editing. All authors have read and approved the final version of the paper submitted to the journal.
